# Electrostatic Spray Drying of a Milk Protein Matrix—Impact on Maillard Reactions

**DOI:** 10.3390/molecules29245994

**Published:** 2024-12-19

**Authors:** Doll Chutani, Todor Vasiljevic, Thom Huppertz, Eoin Murphy

**Affiliations:** 1 Food Chemistry and Technology, Teagasc Food Research Centre, Moorepark, Fermoy, Co., P61 C996 Cork, Ireland; doll.chutani@teagasc.ie (D.C.); eoin.murphy@teagasc.ie (E.M.); 2 Food Quality & Design, Agrotechnology & Food Sciences Group, Wageningen University & Research, 6708 WG Wageningen, The Netherlands; 3 Advanced Food Systems Research Unit, Institute for Sustainable Industries and Liveable Cities, College of Sport, Health and Engineering, Victoria University, Melbourne, VIC 8001, Australia; todor.vasiljevic@vu.edu.au; 4 FrieslandCampina, 3818 LE Amersfoort, The Netherlands; 5 School of Food and Nutritional Sciences, University College Cork, T12 YN60 Cork, Ireland

**Keywords:** electrostatic spray drying, freeze-drying, spray drying, Maillard reactions, reaction kinetics, free -NH_2_-groups, 5-hydroxymethylfurfural

## Abstract

Electrostatic spray drying (ESD) of a milk protein matrix comprising whey protein isolate (WPI), skim milk powder (SMP), and lactose was compared to conventional spray drying (CSD) and freeze-drying (FD). ESD and CSD were used to produce powders at low (0.12–0.14), medium (0.16–0.17), and high (0.31–0.36) levels of water activity (a_w_), while FD powders targeted low a_w_ (0.12). Maillard reaction indicators were studied after drying and during storage for up to 28 days at 20, 40, or 60 °C by measuring free -NH_2_ groups, as an indicator of available lysine, and 5-hydroxymethylfurfural (HMF). After drying, levels of residual free -NH_2_ groups were ~15% higher in ESD and FD powders than in their CSD counterparts. CSD powders also had ~14% higher HMF concentrations compared to their ESD and FD counterparts. Storage led to reductions in free -NH_2_ groups and increases in HMF content in all powders, the extent of which increased with increasing storage temperature. Reductions in free -NH_2_ groups followed first-order reaction kinetics at 20 and 40 °C but second-order reaction kinetics at 60 °C. Lactose crystallization was detected in high-a_w_ CSD powders after 14 d at 40 °C and in both CSD and ESD powders after 7 d at 60 °C. Overall, we found that ESD is a gentle drying technology which enables production of powders with lower Maillard reaction markers.

## 1. Introduction

The Maillard reaction is a non-enzymatic browning reaction that occurs between reducing sugars and amino groups and can significantly affect the quality of food products during processing and storage. The first stage of the Maillard reaction is often referred to as protein glycation, whereas later stages of the Maillard reaction lead to the formation of so-called advanced glycation end-products (AGEs) [1]. The Maillard reaction can affect the sensory attributes of foods, such as color and flavor, but also affect essential nutrients, like the indispensable amino acid lysine. In infant formula, for example, the glycation of lysine reduces the nutritional value of the product, and the formation of potentially harmful compounds such as furfurals and AGEs may raise safety concerns [1].

The Maillard reaction is influenced by many factors, including temperature, water activity (a_w_), pH, the concentrations of lysine and reducing sugars, and the type of reducing sugars [2]. High temperatures during drying processes can also induce protein glycation [3]. Therefore, selecting appropriate drying methods and drying conditions is essential for preventing or minimizing protein glycation and further Maillard reactions. Freeze-drying (FD) is a well-established method for preserving heat-sensitive nutrients. This low-temperature drying technique involves the sublimation of water from frozen materials under low pressure, which minimizes thermal damage [4]. While it is effective at preserving nutrients, FD is both time-consuming and costly, making it less suitable for large-scale operations [5,6]. In contrast, conventional spray drying (CSD) is widely used for its cost-effectiveness and operational scale but exposes products to higher temperatures and shear forces, which can negatively affect heat-labile nutrients such as proteins and vitamins [7,8,9]. Electrostatic spray drying (ESD) is an emerging technology that offers drying at lower temperatures in the presence of an electrostatic field, thus offering options to preserve a product’s nutritional integrity [10,11].

ESD is a low-temperature drying method where a liquid feed can be dried into a powder at exhaust temperatures as low as 30 °C, which is significantly lower than the typical outlet temperatures of 70–90 °C used in CSD methods. In ESD, an electrostatic charge is applied to the feed during atomization using an electrostatic nozzle. This charge is purported to result in the stratification of components in the droplet based on their chemical polarity. The most polar component of the feed (typically water) is driven towards the outer surface, whereas the least polar components tend to remain at the core [10]. This stratification allows the feed to dry at lower temperatures [10]. Recent research highlights several advantages of ESD in encapsulation [12,13], retention of bioactive compounds [14], preservation of microorganisms [15,16], stabilization of volatile flavor compounds [17], and enhancement of oxidative stability [18]. Furthermore, ESD has shown great potential for drying thermosensitive formulations [9,13,18]. Despite its promising capabilities, especially in areas such as the preservation of heat-sensitive materials, only a few studies have explored the application of ESD to dairy proteins, indicating the need for investigation [18].

Storage temperature is another key factor that influences the Maillard reaction. Higher storage temperatures provide the activation energy necessary for the Maillard reaction to proceed more rapidly [2]. This leads to increased formation of browning compounds and AGEs. In contrast, lower storage temperatures tend to slow down these reactions, preserving the nutritional and sensory quality of the product. However, the Maillard reaction still occurs over prolonged storage periods at ambient temperatures, particularly in food matrices with intermediate water activity levels [2].

The objective of this study was to evaluate the effects of different drying methods—ESD, CSD, and FD—on the Maillard reactions in a milk protein matrix comprising caseins, whey proteins, and lactose. The primary focus of this research was to assess the retention of available lysine, measured by changes in free -NH_2_ groups, and the formation of 5-hydroxymethylfurfural (HMF) during drying and storage. The kinetics of these changes during storage as a function of storage temperature and a_w_ were also investigated.

## 2. Results and Discussion

### 2.1. Water Activity

Water activity (a_w_) values of powders produced by ESD, CSD, and FD are presented in Table 1. The values lie within the targeted range and were achieved by using different inlet and outlet temperatures, as described in Section 3.3.

### 2.2. Effect of Drying Techniques on Free Amino Groups

**Figure 1:**
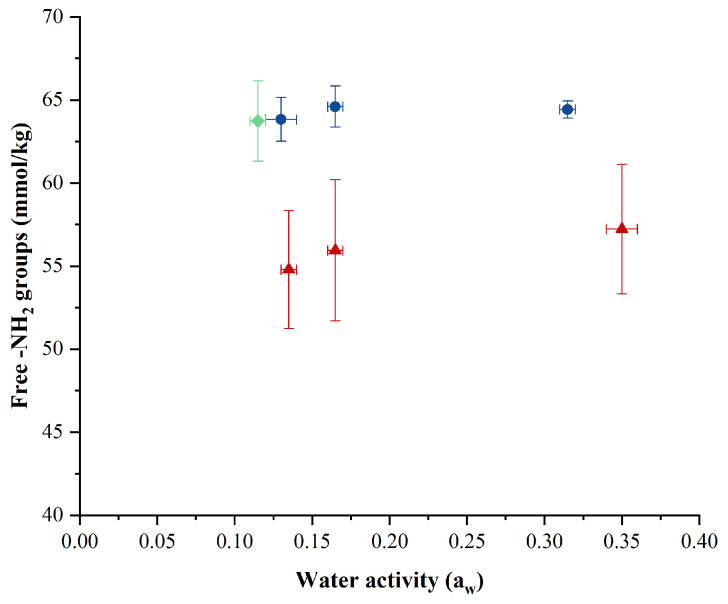
Impact of electrostatic spray drying (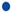), conventional spray drying (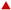), and freeze-drying (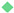) on concentrations of free -NH_2_ groups when powders were produced at different water activity levels. The error bars represent the standard deviations from two experimental trials of each drying condition.

An early consequence of the Maillard reaction is the loss of available lysine because of the reaction between the *ε*-NH_2_ group of lysine with a reducing carbohydrate. Such effects were monitored via the determination of the levels of free -NH_2_ groups. Figure 1 shows that the levels of free -NH_2_ groups immediately after drying were significantly (*p* < 0.05) affected by the drying method employed. Powders prepared with ESD and FD exhibited significantly (*p* < 0.05) higher levels of free -NH_2_ groups (~63–64 mmol/kg powder) across all a_w_ levels compared to CSD powders. Levels of free -NH_2_ groups in low, medium, and high-a_w_ CSD powders were 14%, 13%, and 11% lower, respectively, than those for ESD powders at similar a_w_. The higher levels of residual free -NH_2_ groups in ESD and FD powders can be linked to the low temperature profiles associated with these drying technologies. In contrast, the higher thermal load imposed on the milk protein matrix during the CSD process likely contributed to the loss of free -NH_2_ groups. The type of drying technology can also have a significant impact, as observed by Aalaei et al. [3], who found that FD had the smallest effect on free -NH_2_ groups during skim milk powder production, followed by CSD and then drum drying. A similar study conducted on hempseed protein isolate dried using FD and CSD methods corroborated these findings, with CSD powders showing lower levels of residual free -NH_2_ groups compared to FD and undried products [19]. High drying temperatures or high heat treatments that result in significant blockage of free -NH_2_ groups have been consistently demonstrated in various studies [20,21,22,23]. Although research on the use of ESD for dairy products in this context is limited, lower temperatures in ESD have been shown to be beneficial for the preservation of *Lacticaseibacillus rhamnosus* GG cell viability during drying and storage, which is often compromised by CSD [15].

### 2.3. Effect of Drying Techniques on HMF Concentrations

**Figure 2:**
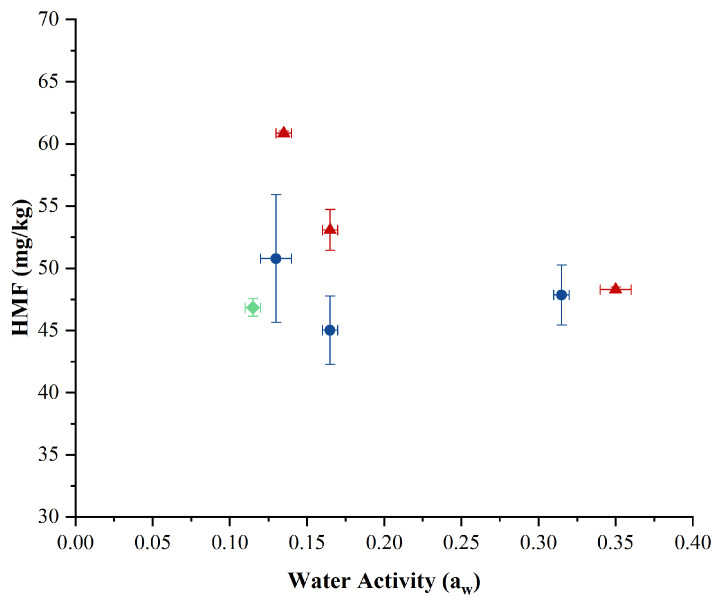
Impact of electrostatic spray drying (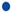), conventional spray drying (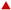), and freeze-drying (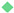) on HMF (5-hydroxymethylfurfural) concentrations when powders were produced at different water activity levels. The error bars represent the standard deviations from two experimental trials of each drying condition.

The products of the Maillard reaction are complex, among which 5-hydroxymethyl furfural (HMF) serves as a characteristic intermediate reaction product [24]. Figure 2 shows the relationship between a_w_ and HMF concentration in powders produced by ESD, CSD, and FD. The results indicate that CSD powders (except those with high a_w_) had higher HMF concentrations than those prepared by ESD and FD. The highest HMF concentration (60 mg/kg powder) was found in the low-a_w_ (0.13) sample produced by CSD samples (Figure 2). This high level is attributed to the higher temperatures required to achieve low a_w_ levels in CSD. As a_w_ increased, the HMF concentration in CSD powders decreased. ESD samples maintained relatively constant HMF levels across all a_w_ values, which were comparable to the level found in FD samples (46 mg/kg). Given that FD does not involve thermal input, the presence of HMF in FD samples likely originates from the SMP and WPI used rather than from HMF being formed during FD. The fact that the ESD process, which is also performed at relatively low temperatures, shows comparable levels of HMF to FD (Figure 2) supports the suggestion that the temperature profiles play a crucial role in the formation of HMF during drying. Masum et al. [13] compared ESD and CSD for the drying of whole milk powder (WMP), SMP, and infant formula (IF) and found that HMF content was lower in ESD powders (90 °C inlet and 35 °C outlet air temperature) than in CSD powders (180 °C inlet and 90 °C outlet air temperature). These authors reported that free HMF was approximately 33% lower in WMP, 57% lower in SMP, and 11% lower in IF produced by ESD compared to CSD [13].

### 2.4. Effect of Powder Storage on Free Amino Groups

**Figure 3:**
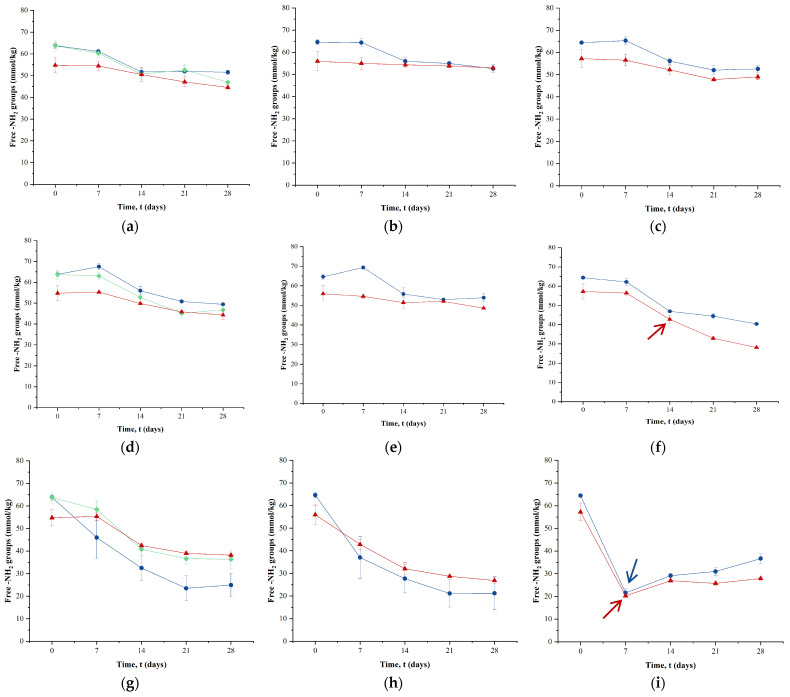
Levels of free -NH_2_ groups in low- (**a**,**d**,**g**), medium- (**b**,**e**,**h**), or high- (**c**,**f**,**i**) water-activity powders produced by electrostatic spray drying (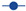), conventional spray drying (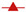), and freeze-drying (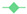) as a function of storage time at 20 (**a**–**c**), 40 (**d**–**f**), or 60 (**g**–**i**) °C. The arrows (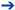: ESD and 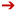: CSD) indicate the time point at which crystallization was detected in the sample via FTIR. FD samples exclusively contained samples with low a_w_. The error bars represent the standard deviations from two experimental trials of each drying condition.

The effect of storage temperature on the free -NH_2_ groups in the powdered milk protein matrix prepared using ESD, CSD, and FD is shown in Figure 3. Free -NH_2_ groups showed small changes over time at 20 °C in all samples (Figure 3a–c). While powders produced by ESD and FD had higher levels of residual free -NH_2_ groups than CSD powders immediately after drying, there were no significant differences (*p* > 0.05) between drying techniques after 28 d of storage at 20 °C. However, a significant difference (*p* < 0.05) was found between low- and medium-a_w_ samples, with low-a_w_ samples showing lower residual -NH_2_ groups compared to medium-a_w_ samples after 28 d at 20 °C. The lower levels of free -NH_2_ groups in the low-a_w_ CSD samples after storage are due to their lower levels in the beginning. High-a_w_ samples stored for 28 d at 20 °C did not differ significantly from low- and medium-a_w_ samples in terms of levels of residual free -NH_2_ groups.

When powders were stored at 40 °C, there was a significant (*p* < 0.05) reduction in free -NH_2_ groups compared to storage at 20 °C. This finding is consistent with data from Higgs et al. [25], who reported a negligible loss of free -NH_2_ groups (reported as available lysine) in whey protein concentrate during storage at 30 °C but severe losses at 40 °C. Despite the elevated storage temperature, ESD and FD powders retained significantly (*p* < 0.05) higher levels of free -NH_2_ groups than CSD powders. After 28 d at 40 °C, the levels of free -NH_2_ groups in ESD powders were 49 mmol/kg for low a_w_, 54 mmol/kg for medium a_w_, and 40 mmol/kg for high a_w_. In comparison, the CSD powders contained 44, 49, and 28 mmol of free -NH_2_ groups per kg for low, medium, and high a_w_ levels, respectively. CSD powders with high a_w_ (0.35) exhibited lactose crystallization and higher losses of free -NH_2_ groups than equivalent ESD powders with an a_w_ level of 0.32 when stored at 40 °C, as can be seen in Figure 3f. FD powders, which were essentially low-a_w_ samples, retained 47 mmol/kg of -NH_2_ groups after 28 d, comparable to the values observed during storage at 20 °C.

During storage at 20 °C and 40 °C, loss of free -NH_2_ groups was found to follow first-order reaction kinetics with significantly (*p* < 0.05) higher rate constants at 40 °C (Table 2). A similar trend was shown by Aalaei et al. [26], when SMP was stored at 30 °C, 32.5 °C, and 35 °C for 30 days and first-order rate constants for the loss of free -NH_2_ groups increased by 2.5-fold at 35 °C compared to 30 °C. At 40 °C, CSD samples with high a_w_ showed the highest loss of -NH_2_ groups, with the rate constant increasing by 1.5-fold compared to the equivalent ESD powder (Table 2). Samples with low and medium a_w_ stored at 20 and 40 °C had similar values of rate constants (Table 2), with the exception of a CSD sample of medium a_w_, which had shown the lowest rate constant value in all cases.

Storing the powders at 60 °C led to higher losses of free -NH_2_ groups across all powders, with higher-order reaction kinetics observed for low- and medium-a_w_ samples compared to storage at 20 or 40 °C. Schmitz-Schug et al. [21] also observed second-order reaction kinetics for the loss of the *ε*-amino group of lysine in a model dairy formulation (lactose/protein ratio of 5:1) when heated at 60–90 °C for 30 min. Second-order reaction kinetics were applied to describe the loss of free -NH_2_ groups (reported as available lysine) from whole milk when heated at 130–160 °C for 50 s to 150 min [27]. The authors noted that second-order kinetics provided a better fit than first-order kinetics for higher temperatures and longer time treatments, which resulted in greater lysine losses. The goodness of fit of the two models, i.e., first- and second-order, was also evaluated by residual analysis and an *F*-test, as detailed in Section S1 of Supplementary Materials. ESD powders showed higher rate constant values than CSD powders at 60 °C for low- and medium-a_w_ samples. For powders with high a_w_ levels, no significant kinetic model could be fitted, likely because the loss occurred too quickly to be detected with the available data points.

As shown in Figure 3i, high-a_w_ samples of ESD and CSD powders showed a sharp decrease in the concentration of free -NH_2_ groups within the first 7 days of storage at 60 °C, dropping to approximately 20–21 mmol/kg. Both powders showed lactose crystallization at this point, as indicated by arrows in Figure 3i. Medium-a_w_ samples also underwent substantial losses in free -NH_2_ groups at 60 °C, with final concentrations of 21–26 mmol/kg after 28 d. In contrast, low-a_w_ samples retained more free -NH_2_ groups, with CSD powders retaining 38.2 mmol/kg, followed by FD powders at 36.3 mmol/kg, and ESD powders with the lowest retention at 24.9 mmol/kg. A similar effect was observed in a study by Ford et al. [28], who found that 50% of amino groups in lysine in SMPs were blocked after 2 weeks of storage at 70 °C, and from 3 weeks onward, it was even more pronounced. Although water activity levels were not reported in that study, the progression of the Maillard reaction is known to be significantly influenced by storage temperature [1,2,25,26,29].

### 2.5. Effect of Powder Storage on HMF Concentrations

**Figure 4:**
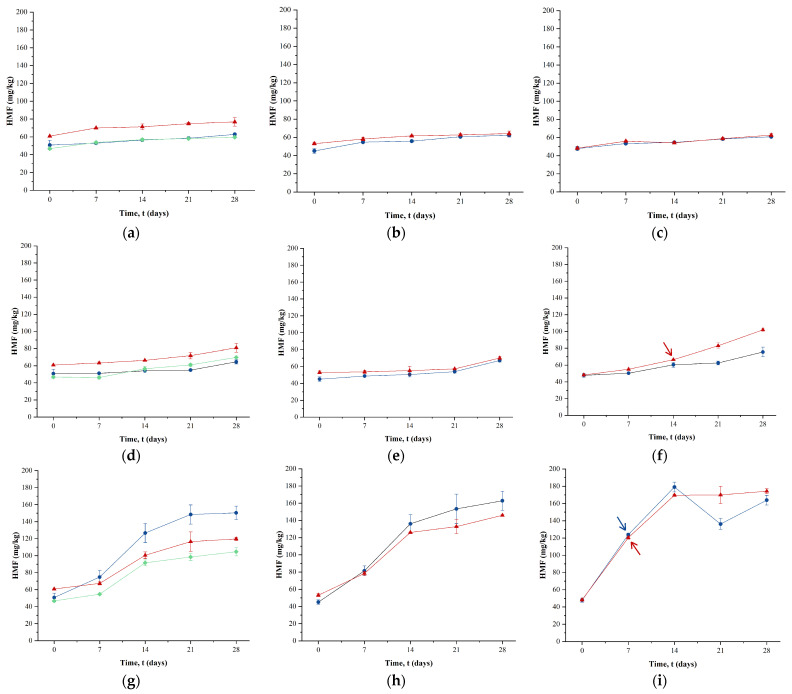
Levels of HMF in low- (**a**,**d**,**g**), medium- (**b**,**e**,**h**) or high- (**c**,**f**,**i**) water-activity powders produced by electrostatic spray drying (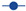), conventional spray drying (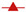), and freeze-drying (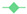) as a function of storage time at 20 (**a**–**c**), 40 (**d**–**f**), or 60 (**g**–**i**) °C. The arrows (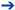: ESD and 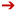: CSD) indicate the time point at which crystallization was detected in the sample via FTIR. FD samples exclusively contained samples with low a_w_. The error bars represent the standard deviations from two experimental trials of each drying condition.

HMF concentration increased in all the samples following 28 days of storage at 20 °C (see Figure 4). By day 28, the HMF levels in the ESD powders reached 62 mg/kg for low- and medium-a_w_ powders and 60 mg/kg for high-a_w_ powders. FD powders showed an increase in HMF from 47 mg/kg at day 0 to 60 mg/kg after 28 days of storage at 20 °C. In comparison, CSD powders showed significantly higher increases (*p* < 0.05) than both ESD and FD, with values reaching 77 mg/kg, 64 mg/kg, and 63 mg/kg for low, medium, and high-a_w_ powders, respectively. Storage has been shown to negatively influence the Maillard reaction in powdered milk or infant formula. For example, when whole milk powder was stored at 25 °C, a significant increase in HMF levels was observed [29]. Similarly, HMF levels in nine different infant formula powders and three whole milk powders increased significantly during 9 months of storage at room temperature [30].

Storage at 40 °C led to a significantly (*p* < 0.05) higher increase in HMF concentration across all samples compared to storage at 20 °C. The highest concentration was found in the powders prepared by CSD with high a_w_, reaching 102 mg/kg after 28 days of storage (Figure 4f). The CSD powders with low and medium a_w_ also showed considerable increases in HMF concentration (Figure 4d,e), albeit to a lesser extent than the high a_w_ level. An increase in HMF concentration during storage was also seen in the ESD and FD powders, but the extent of HMF formation at 40 °C was significantly (*p* < 0.05) lower than in the CSD powders at the same temperature.

After storage at 60 °C, HMF levels reached approximately 136–145 mg/kg in medium-a_w_ samples after 28 days, while high-a_w_ samples attained similar HMF levels within 14 days for both ESD and CSD powders. The statistical analysis results indicated no significant difference (*p* > 0.05) between ESD and CSD in HMF levels at day 28 when stored at 60 °C; however, both were significantly different (*p* < 0.05) compared to FD. This indicates that, while differences in HMF formation between ESD and CSD were evident at lower storage temperatures, these differences diminished at higher temperatures, e.g., at 60 °C.

The rate of increase of HMF followed first-order kinetics at all storage temperatures and a_w_. The rate constant values along with other kinetic parameters are presented in Table 3. Under all conditions, the rate constants for HMF formation during storage at 60 °C were significantly (*p* < 0.05) higher than those observed at 20 °C and 40 °C, indicating that higher temperatures significantly accelerate the reaction kinetics (see Table 3). A similar trend, as observed in the rate of loss of free -NH_2_ groups, was found with variations in a_w_ at 20 °C and 40 °C, where the rate constant values for low- and medium-a_w_ samples were generally comparable, except for the medium-a_w_ sample in the CSD method. Furthermore, at 40 °C, the rate constant for high-a_w_ samples processed by CSD was greater than that of the ESD samples. Similar kinetics work has been reported by Baldwin et al. [31] for whey protein concentrate powders stored at different a_w_ and temperatures. For powders with an a_w_ of 0.33, the Maillard reaction rate (indicated by the formation of lactulosyl-lysine) was reported to increase significantly as the temperature rose from 30 °C to 40 °C. Their study also explored a_w_ levels above 0.33 and found that the Maillard reaction rate increases with higher a_w_ levels. Le et al. [32] also reported that HMF levels in milk protein concentrate powders showed little change when stored at 25 °C and gradually increased with increasing storage temperature to 40 °C.

### 2.6. Correlations

A strong negative correlation (Pearson’s R = −0.89) was observed between the loss of free -NH_2_ groups and the formation of HMF in ESD, CSD, and FD powders with low, medium, and high a_w_ levels and stored at temperatures of 20 °C, 40 °C, or 60 °C. This indicates that as the HMF concentration increased, levels of free -NH_2_ groups decreased. When crystallized samples, which were detected by FTIR spectroscopy (see Supplementary Materials), were removed from the correlation analysis (a total of 24 samples out of 182), the correlation increased to −0.92, indicative of the unpredictable effect of crystallization on the Maillard reactions. Indeed, the behavior of samples at 60 °C, i.e., the transition from first- to second-order kinetics for lysine (free -NH_2_ groups) blockage, is indicative of a fundamental shift in the reaction mechanism even before crystallization. This suggests that samples stored at 60 °C may not accurately represent the long-term storage behavior due to (a) crystallization, which would not typically happen during prolonged storage at lower temperatures, and (b) changes in the Maillard reaction mechanism.

## 3. Materials and Methods

### 3.1. Materials

Whey protein isolate (WPI) was obtained from FrieslandCampina (Amersfoort, The Netherlands). Lactose was obtained from Tirlán (Ballyragget, Ireland). Skim milk powder (SMP) was procured from Dairygold (Mitchelstown, Ireland), and 1 N Sodium hydroxide (NaOH), o-phthaldialdehyde (OPA), N-acetyl-L-cysteine (NAC), sodium dodecylsulfate (SDS), absolute ethanol, orthoboric acid, oxalic acid, trichloroacetic acid (TCA), and tert-Butyl alcohol (TBA) were purchased from Sigma-Aldrich (Melbourne, Australia). All the chemicals and reagents used in this study were of analytical grade.

### 3.2. Sample Preparation

A dispersion of 30% *w*/*w* solids was prepared with a protein-to-carbohydrate ratio of 1:7.2, using 30.68 g of whey protein isolate (WPI), 23.86 g of skim milk powder (SMP), and 249 g of lactose to prepare a batch of 1 kg, with the remainder being made up of water. The ratio of whey protein to casein was set to 80:20. The pH of the dispersion was adjusted to 6.8 using 1 N NaOH [33].

### 3.3. Drying Processes

#### 3.3.1. Electrostatic Spray Drying

ESD was performed using a laboratory-scale electrostatic spray dryer (PolarDry^®^ Model 001, Chicago, IL, USA). The powders were produced in duplicate at three target a_w_ levels: low a_w_ (0.10–0.15); medium a_w_ (0.16–0.20); and high a_w_ (>0.3). The drying parameters were kept constant during production: nitrogen flow rate of 15 m^3^/h, atomizing gas pressure of 150 kPa, atomizing gas temperature of 35 °C, and electric voltage of 5 kV. Inlet air temperatures were set to 100 °C for low-a_w_ samples and 90 °C for medium- and high-a_w_ samples. The feed rate was adjusted accordingly to achieve the required a_w_; this corresponded to outlet temperatures of 42 °C (low a_w_), 38 °C (medium a_w_), and 35 °C (high a_w_). The feedstock temperature was kept at 35 °C.

#### 3.3.2. Conventional Spray Drying

CSD was conducted using a pilot-scale spray dryer (YC–018, Shanghai Pilotech Instrument & Equipment Co., Ltd., Shanghai, China). The powders were produced in duplicate at three target water activity levels, similar to ESD: low a_w_ (0.10–0.15), medium a_w_ (0.16–0.20), and high a_w_ (>0.3), with a constant airflow rate of 34 m^3^/h. The operational conditions were as follows: inlet and outlet air temperatures of 165 °C and 105 °C for low a_w_; 145 °C and 85 °C for medium a_w_; and 145 °C and 70 °C for high a_w_, respectively. The feedstock temperature was kept at 35 °C.

#### 3.3.3. Freeze-Drying

The dispersions were frozen at −20 °C for 24 h and subsequently dried in an Alpha 1–4 LSCbasic freeze-dryer (Martin Christ, Osterode am Harz, Germany). A temperature of −50 °C was used for both drying stages. The freeze-dried powder was produced at a condenser temperature of −50 °C and a pressure of 1 mbar for 50 h in duplicate at a targeted low a_w_ level (0.10–0.15).

### 3.4. Measurement of Water Activity

The water activity values of the powders were measured after production using a portable AquaLab Paw Kit (Graintec, Wendouree, Australia), with each analysis being performed in duplicate.

### 3.5. Shelf Life Study

**Figure 5:**
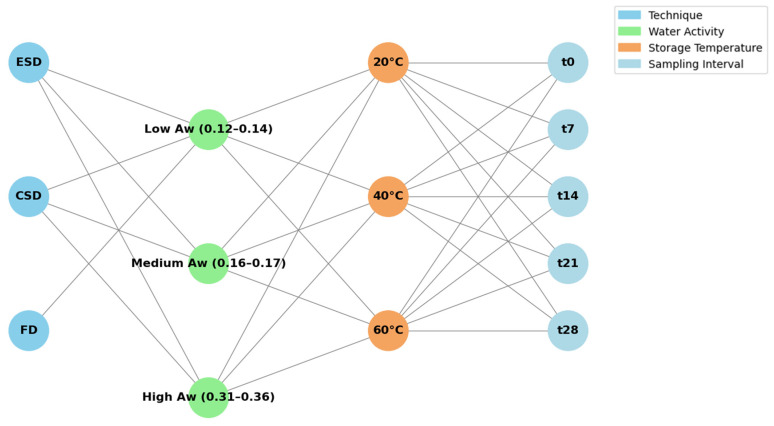
Graphical representation of the experimental design of this study.

To determine changes in the powders during storage, they were stored in polypropylene tubes, and the tubes were sealed in aluminum foil bags. Samples were stored in an oven at 20 °C, 40 °C, or 60 °C immediately after production for up to 28 days. Samples were collected every 7 days and stored in a freezer (−18 °C) until further analysis. A graphical representation of the design of this study can be found in Figure 5.

#### 3.5.1. Measurement of Free -NH_2_ Groups

Free -NH_2_ groups were measured using the o-phthaldialdehyde/N-acetyl-L-cysteine (OPA/NAC) spectrophotometric method, as described by Vigo et al. [34]. Samples (10 mg) were dissolved in 1 mL of 5% (*w*/*w*) sodium dodecylsulfate (SDS) solution. An OPA-NAC reagent was prepared by mixing 25 mL of 0.05 M ethanolic OPA solution, 25 mL of 0.05 M aqueous NAC solution, and 200 mL of 0.02 M boric acid-borate buffer solution (pH 9.5), and water to obtain a total volume of 1 L. A 2.5 mL aliquot of this reagent was mixed with 0.5 mL of the sample solution at room temperature, and the mixture was diluted with water to obtain a final volume of 6.25 mL. Using a spectrophotometer, absorbance was measured at 335 nm within 10–15 min after dilution (Biochrom Libra S12 UV/Vis, Biochrom, Cambridge, UK). The coefficient of variation for this assay was <3%. The concentration of free -NH_2_ groups was calculated from a standard curve constructed using L-lysine standards.

#### 3.5.2. Measurement of HMF Concentration

HMF concentration was determined using a method described previously [35,36]. The powder sample (0.2 g) was added to 2 mL of 0.3 M oxalic acid solution, mixed thoroughly, and placed in a boiling water bath for 1 h, after which it was removed and cooled to room temperature using cold water. Trichloroacetic acid solution (40%, *w*/*v*, 2 mL) was added, and the mixture was allowed to stand for 15 min after shaking. Then, the mixture was centrifuged at 4000× *g* for 15 min at room temperature, followed by filtration of the supernatant using Whatman Filter Paper No. 4 (Sigma-Aldrich, Melbourne, Australia). Subsequently, 0.2 mL of 0.05 M TBA was added to the supernatant, and this mixture was kept in a water bath at 45 °C for 30 min. Absorbance was measured at 443 nm with a spectrophotometer (Biochrom) after removing the mixture from the water bath and cooling it to room temperature.

#### 3.5.3. Kinetics of the Maillard Reaction

The kinetics of the Maillard reaction were monitored through the loss of free -NH_2_ groups and the generation of HMF in the powders. First- and second-order rate constants for the loss of free -NH_2_ groups or formation of HMF, along with their standard errors, were calculated using linear regression analysis. The equations used were either (i) ln[A_t_] − ln[A_0_] = −kt for first-order kinetics or (ii) 1/[A_t_] = 1/[A_0_] + kt for second-order kinetics, where A_t_ represents the concentration of free -NH_2_ groups or HMF at time t, A_0_ represents the concentration of free -NH_2_ groups or HMF at t = 0, and k represents the rate constant [37].

A two-way analysis of variance (ANOVA) was performed to assess statistical differences across the levels of free -NH_2_ groups and HMF, with a 95% confidence interval. Pearson’s correlation coefficient was calculated to examine the relationship between them. Additionally, ANOVA was applied to determine whether the rate constants significantly differed from zero. To further evaluate the validity of the linear equations, an F-test was used to check for a lack of fit. These statistical tests ensured the reliability of the kinetic models applied to the data. Data analysis was performed using Python v3.10.12 (The Python Software Foundation, Wilmington, DE, USA), specifically employing libraries such as NumPy, SciPy, and Matplotlib.

### 3.6. Fourier Transform Infrared Spectroscopy Measurements

**Figure 6:**
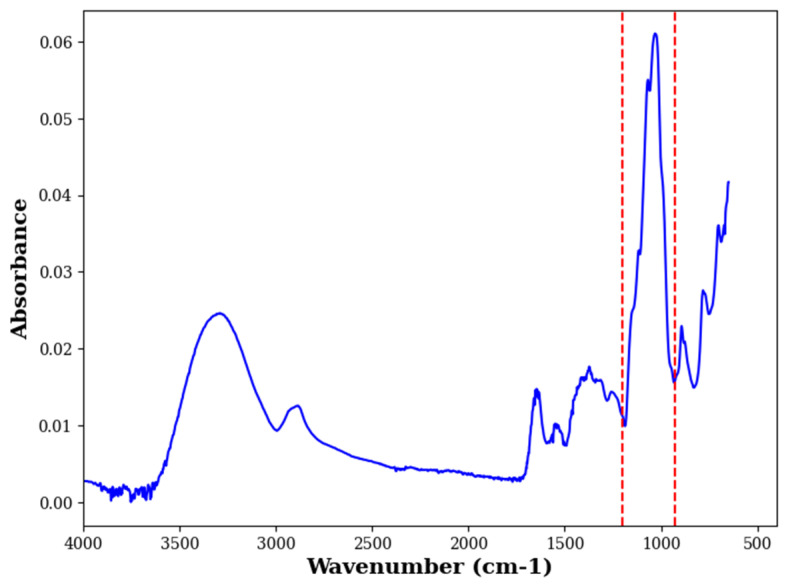
FTIR spectra of (ESD) powder at time t = 0 of storage in the range 4000–400 cm^−1^ with the annotated region of interest indicated between the red dashed lines. FTIR—Fourier Transform Infrared and ESD—electrostatic spray drying.

Infrared spectra of powder samples at each time point and storage temperature were obtained using a PerkinElmer Fourier transform infrared (FTIR) spectrometer (Frontier, PerkinElmer, Shelton, CT, USA). For each spectrum, an average of 16 scans were recorded in the range of 4000 to 400 cm^−1^ with a 4 cm^−1^ resolution. Atmospheric background spectra were scanned at the beginning of the measurements with a blank Diamond ATR cell using the same instrumental conditions as for the acquisition of sample spectra and were subtracted automatically. The spectra were obtained in absorbance mode in triplicate for each sample, resulting in an average of six spectra per sample condition. Spectral data were processed using functions such as atmospheric compensation and vector normalization. Differences in spectra were analyzed to identify lactose crystallization based on the wavenumber region 1200–900 cm^−1^ (Figure 6), as described previously [24,38,39]. Results on lactose crystallization are provided in Section S2 of the Supplementary Materials.

## 4. Conclusions

In ESD, the application of an electric charge to liquid feed materials enhances the stability and functionality of products. This was shown in this study, where this technology was compared to established drying processes such as FD and CSD to produce milk protein matrices. Lower processing temperatures in ESD during drying resulted in powders that were comparable to those produced by FD in terms of Maillard reaction indicators—free *ε*-NH_2_ groups and HMF. However, this study also demonstrates that storage at higher temperatures and higher a_w_ will rapidly counter the beneficial effect of ESD and may lead to undesirable changes, such as lactose crystallization in the product. Therefore, product water activities and storage temperatures should be carefully chosen to limit the rate of Maillard reactions during storage, which can drastically counteract any potential benefit arising from ESD technology. While these results look promising, further research is necessary to determine the scalability of the findings and to fully explore the benefits of ESD technology for dairy matrices as well as other thermosensitive biological products.

## Figures and Tables

**Table 1:** Water activity values of powders produced by different drying techniques (ESD, CSD, and FD) at different drying temperatures.

Drying Technique	Drying Parameters		Water Activity (a_w_)
	Inlet Temperature (°C)	Outlet Temperature (°C)	
ESD	100	42	0.13 ± 0.01
	90	38	0.17 ± 0.01
	90	35	0.32 ± 0.01
CSD	165	105	0.14 ± 0.01
	145	85	0.17 ± 0.01
	145	70	0.35 ± 0.01
FD	n/a	n/a	0.12 ± 0.01

ESD—electrostatic spray drying; CSD—conventional spray drying; FD—freeze-drying; and n/a—not applicable (*n* = 2, mean ± SD; standard deviation from two experimental trials of each drying condition).

**Table 2:** Comparison of the rate of loss of free -NH_2_ groups in powders produced with different water activity levels using different drying techniques (ESD, CSD, and FD) when stored at 20 °C, 40 °C, or 60 °C for a 4-week period: Rate constants, model equations, and statistical parameters.

Drying Technique	Storage Temperature (°C)	Water Activity (a_w_)	Slope (Rate Constant, k (day^−1^))	Model Equation	R-Squared	RMSE	F- Statistic	F-Statistic *p*-Value
ESD	20	Low	0.0084	A_t_ = 9.17 e^−0.0084t^	0.80	0.054	11.85	0.041
		Medium	0.0081	A_t_ = 9.55 e^−0.0081t^	0.90	0.035	26.11	0.015
		High	0.0091	A_t_ = 9.59 e^−0.0091t^	0.84	0.049	16.27	0.027
	40	Low	0.0114	A_t_ = 9.78 e^−0.0114t^	0.84	0.063	15.83	0.028
		Medium	0.0090	A_t_ = 9.78 e^−0.0090t^	0.70	0.076	6.88	0.079
		High	0.0182	A_t_ = 9.57 e^−0.0182t^	0.93	0.066	37.68	0.009
	60	Low	0.0068	1/A_t_ = 0.111 + 0.0068t	0.91	0.027	30.28	0.011
		Medium	0.0082	1/A_t_ = 0.122 + 0.0082t	0.94	0.027	46.42	0.006
		High	No model could be fitted					
CSD	20	Low	0.0080	A_t_ = 8.19 e^−0.0080t^	0.96	0.021	68.74	0.004
		Medium	0.0019	A_t_ = 8.16 e^−0.0019t^	0.99	0.002	332.82	0.000
		High	0.0068	A_t_ = 8.43 e^−0.0068t^	0.86	0.035	18.72	0.023
	40	Low	0.0088	A_t_ = 8.23 e^−0.0088t^	0.92	0.032	36.27	0.009
		Medium	0.0047	A_t_ = 8.19 e^−0.0047t^	0.90	0.020	28.46	0.013
		High	0.0280	A_t_ = 9.04 e^−0.0280t^	0.95	0.080	59.98	0.004
	60	Low	0.0023	1/A_t_ = 0.121 + 0.0023t	0.88	0.011	22.76	0.018
		Medium	0.0049	1/A_t_ = 0.129 + 0.0049t	0.96	0.013	73.77	0.003
		High	No model could be fitted					
FD	20	Low	0.0107	A_t_ = 9.25 e^−0.0107t^	0.88	0.050	22.06	0.018
	40	Low	0.0136	A_t_ = 9.50 e^−0.0136t^	0.87	0.068	19.50	0.022
	60	Low	0.0033	1/A_t_ = 0.107 + 0.0033t	0.90	0.014	26.20	0.014

FD samples exclusively contained samples with low a_w_. ESD—electrostatic spray drying; CSD—conventional spray drying; FD—freeze-drying; A_t_ = concentration at time t; t = time; and RMSE = Root Mean Square Error.

**Table 3:** Comparison of the rate of formation of HMF in powders produced with different water activity levels using different drying techniques (ESD, CSD, and FD) when stored at 20 °C, 40 °C, or 60 °C for a 4-week period: Rate constants, model equations, and statistical parameters.

Drying Technique	Storage Temperature (°C)	Water Activity (a_w_)	Slope (Rate Constant, k (day^−1^))	Model Equation	R-Squared	RMSE	F-Statistic	F-Statistic *p*-Value
ESD	20	Low	0.0075	A_t_ = 50.55 e^0.0075t^	0.99	0.0090	338.54	0.0002
		Medium	0.0107	A_t_ = 47.62 e^0.0107t^	0.87	0.0535	19.75	0.0244
		High	0.0082	A_t_ = 48.91 e^0.0082t^	0.96	0.0222	66.61	0.0056
	40	Low	0.0079	A_t_ = 49.15 e^0.0079t^	0.81	0.0493	12.47	0.0301
		Medium	0.0129	A_t_ = 43.92 e^0.0129t^	0.88	0.0598	22.68	0.0148
		High	0.0162	A_t_ = 46.71 e^0.0162t^	0.95	0.0452	62.74	0.0034
	60	Low	0.0408	A_t_ = 57.30 e^0.0408t^	0.89	0.1869	23.36	0.0171
		Medium	0.0458	A_t_ = 55.05 e^0.0458t^	0.87	0.2297	19.47	0.0220
		High	No model could be fitted					
CSD	20	Low	0.0076	A_t_ = 63.44 e^0.0076t^	0.86	0.0387	18.79	0.0242
		Medium	0.0065	A_t_ = 54.62 e^0.0065t^	0.89	0.0287	25.25	0.0179
		High	0.0081	A_t_ = 49.78 e^0.0081t^	0.85	0.0429	17.62	0.0263
	40	Low	0.0100	A_t_ = 59.38 e^0.0100t^	0.95	0.0302	53.18	0.0044
		Medium	0.0089	A_t_ = 50.84 e^0.0089t^	0.74 *	0.0673	8.50	0.0536
		High	0.0272	A_t_ = 46.74 e^0.0272t^	0.99	0.0310	378.60	0.0002
	60	Low	0.0272	A_t_ = 61.21 e^0.0272t^	0.91	0.1086	30.63	0.0118
		Medium	0.0364	A_t_ = 60.26 e^0.0364t^	0.88	0.1703	22.40	0.0181
		High	No model could be fitted					
FD	20	Low	0.0080	A_t_ = 49.05 e^0.0080t^	0.85	0.0432	16.63	0.0308
	40	Low	0.0153	A_t_ = 44.67 e^0.0153t^	0.93	0.0530	40.99	0.0057
	60	Low	0.0314	A_t_ = 48.54 e^0.0314t^	0.88	0.1466	22.43	0.0175

* (*p* > 0.05). FD samples exclusively contained samples with low a_w_. ESD—electrostatic spray drying; CSD—conventional spray drying; FD—freeze-drying; A_t_ = concentration at time t; t = time; and RMSE = Root Mean Square Error.

## Data Availability

Data are contained within the article and Supplementary Materials.

## Supplementary Materials

The following supporting information can be downloaded at: https://www.mdpi.com/article/10.3390/molecules29245994/s1, Section S1: Evaluation of Model Fit; Figure S1: Normal probability plot of the (a) first-order model and (b) second-order model of free -NH_2_ group reduction in ESD powder with medium water activity under storage at 60 °C; Section S2: FTIR Spectroscopy; Figure S2: FTIR spectra of high-a_w_ powders produced by (**a**) ESD and stored at 40 °C; (**b**) CSD and stored at 40 °C; (**c**) ESD and stored at 60 °C; and (**d**) CSD and stored at 60 °C, acquired every 7 days over a 4-week period (t0⎯⎯, t7⎯⎯, t14⎯⎯, t21⎯⎯ and t28⎯⎯). Spectra for low and medium a_w_ are not included due to a lack of observed spectral differences. FTIR—Fourier Transform Infrared, ESD—electrostatic spray drying, CSD—conventional spray drying, and a_w_—water activity.
