# Supplementary material for: Electrostatic Spray Drying of a Milk Protein Matrix—Impact on Maillard Reactions
Source: Molecules. 2024 Dec 19;29(24):5994. doi: 10.3390/molecules29245994 (PMC11676460; doi:10.3390/molecules29245994)
Supplement: Supplementary file 1 [file molecules-29-05994-s001.zip › molecules-3338000-supplementary.pdf]

1. Evaluation of Model Fit

The goodness of fit of the two models i.e. first- and second-order was evaluated by residual analysis and by the *F* test. The normal probability plot of the first and second-order model for the medium a_w ESD powder is exemplarily shown in Figure S1. The data points follow a straight line in Figure S1b indicates that the residuals are normally distributed, i.e., the second order model predicts the data well.

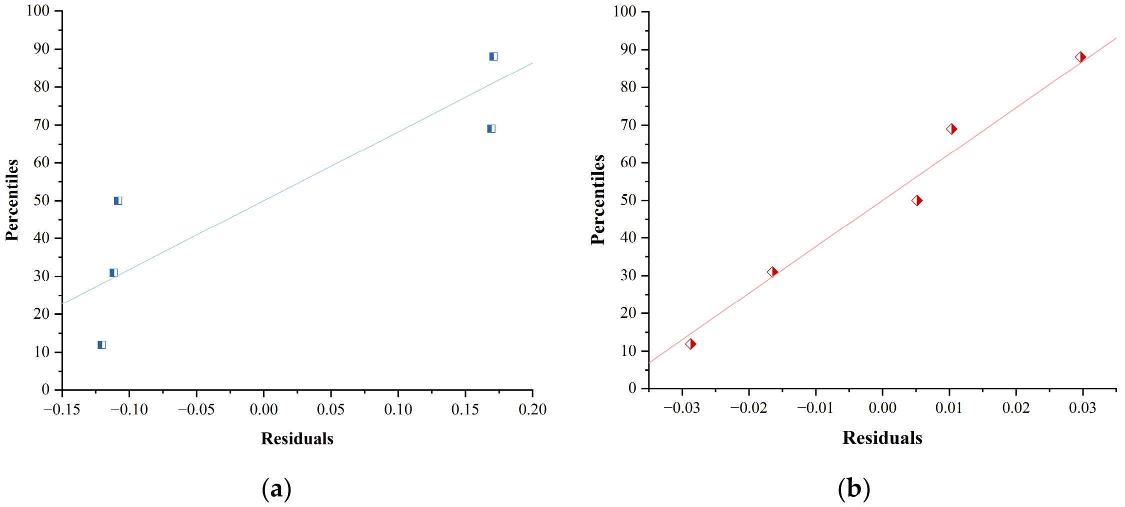

Figure S1. Normal probability plot of the (**a**) first-order model and (**b**) second-order model of free -NH2 group reduction in ESD powder with medium water activity under storage at 60 °C

2. FTIR Spectroscopy

Infrared spectral configurations were recorded for all powders produced by ESD, CSD and FD at low, medium and high a_w levels, subjected to storage at different temperatures of 20°C, 40°C and 60°C. FTIR spectra of high a_w ESD and CSD powders stored at 40°C and 60°C are represented in Figure S2, recorded at different time points from t0 to t28. Low and medium a_w powders did not show any spectral differences throughout the storage period, so their spectra are not shown. Similarly, powders stored at 20°C showed no spectral differences and are therefore also omitted. It can be observed from Figure S2a that the spectral configuration of ESD powder did not change throughout the 28 days storage period at 40°C. However, alterations in the spectral patterns occur at band 1200-900 cm^{-1} occurred at t14, indicating changes in the CSD powder after 14 days of storage at the same temperature (Figure S2b). The changes in band suggest the crystallization of lactose in the CSD powder. Crystallized lactose has sharper peaks in this region compared to amorphous lactose as shown in a study by Yu Lei et al. [24], where he studied FTIR spectral differences in milk powders with added crystallized lactose in different proportions. Milk powder with 30, 40 and 100% crystallized lactose had a small shoulder peak at 3528 $cm^{-1,}$ which was not observed in powders having 0 and 10% of crystallized lactose. Further, in the range 1150 to 1030 cm^{-1}, the peaks of lactose were gradually getting stronger and sharp. Similar observation was reported by the work of Mathlouthi et al. [39] while studying the spectra of amorphous and crystalline sucrose in the range 1200 – 800 cm^{-1}. From Figure S2b, the peaks observed around 1200–900 cm^{-1} at t14 were found to be more defined and sharper compared to t0 or t7. Furthermore, a sharp, distinct, O–H stretch peak at 3524 cm^{-1} is observed and is indicative of constrained water molecules in the crystal lattice [24, 38]. When high a_w powders produced by ESD and CSD were stored at the elevated temperature of 60°C, lactose crystallization, which was observed in SD powders after 14 days of storage at 40°C, occurred earlier—within just 7 days—for both ESD (Figure S2c) and CSD powders (Figure S2d).

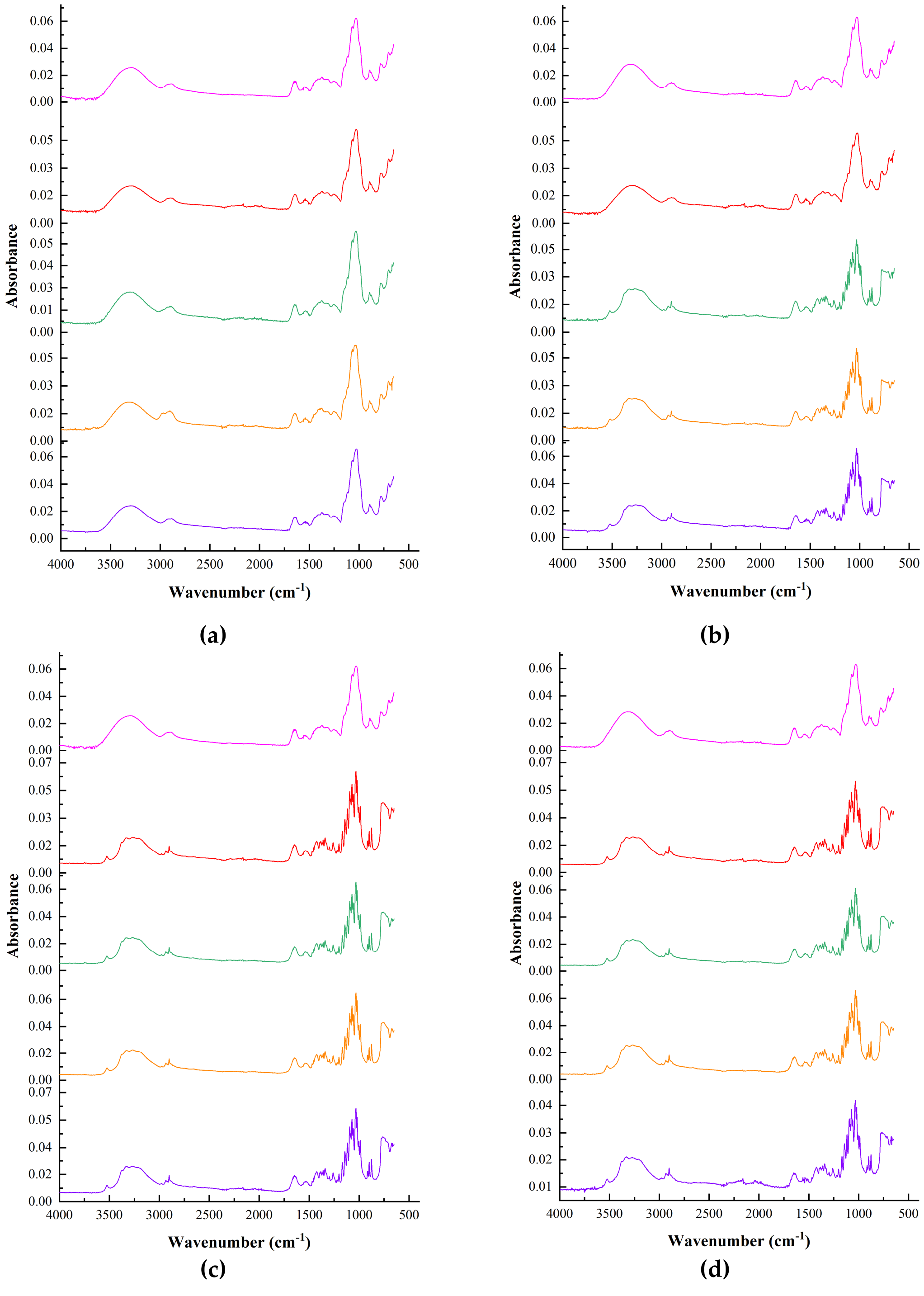

Figure S2. FTIR spectra of high-aw powders produced by (a) ESD and stored at 40 °C; (b) CSD and stored at 40 °C; (c) ESD and stored at 60 °C; and (d) CSD and stored at 60 °C, acquired every 7 days over a 4-week period (t0—, t7—, t14—, t21— and t28—). Spectra for low and medium aw are not included due to a lack of observed spectral differences. FTIR—Fourier Transform Infrared, ESD—electrostatic spray drying, CSD—conventional spray drying, and aw—water activity.
